# The Evaluation of the Relationship Between Oral Habits Prevalence and COVID-19 Pandemic in Adults and Adolescents: A Systematic Review

**DOI:** 10.3389/fpubh.2022.860185

**Published:** 2022-03-04

**Authors:** Amirhossein Mirhashemi, Mohammad Reza Khami, Mohammdjavad Kharazifard, Rashin Bahrami

**Affiliations:** ^1^Department of Orthodontics, School of Dentistry, Tehran University of Medical Sciences, Tehran, Iran; ^2^Research Center for Caries Prevention, Dentistry Research Institute, Community Oral Health Department, School of Dentistry, Tehran University of Medical Sciences, Tehran, Iran; ^3^Tehran University of Medical Sciences, Tehran, Iran; ^4^Departments of Orthodontics, School of Dentistry, Tehran University of Medical Sciences, Tehran, Iran

**Keywords:** COVID-19, SARS-CoV-2, bruxism, adult, adolescent

## Abstract

**Background and Objective:**

The aim of this study was to systematically review the relationship between oral habits (bruxism- temporomandibular disorders) and COVID-19 pandemic in adults and adolescents.

**Method and Material:**

A comprehensive search of the literature through PubMed, Scopus, Embase, google scholar and Cochrane databases was conducted. Such keywords as COVID-19, SARS-CoV-2, bruxism, adult, and adolescent were used.

**Results:**

In the initial search 818 articles were obtained; 68 cases were duplicates and excluded. By reviewing the article title, 714 articles were removed because they were not relevant to the topic. The remaining articles were reviewed, and studies that did not meet the inclusion criteria, as well as letter to editors and expert opinions were excluded. Finally, 11 articles were allowed to enter the study. Out of 11 related articles, 5 studies were excluded from the present study due to mismatch of the target population; and finally 6 articles were thoroughly reviewed.

**Conclusion:**

Studies have shown that stress caused by the COVID-19 pandemic increases detrimental oral habits such as bruxism as well as temporomandibular disorders in adults and adolescents; In general, young single women are at high risk and more exposed to these harmful oral habits.

## Introduction

Bruxism is defined as an unconscious oral habit of rhythmical, unfunctional clenching, grinding, and making chewy sounds with the teeth while making movements that are not part of the masticatory function and that lead to occlusal trauma (1). Bruxism is one of the major risk factors for temporomandibular disorders (TMD) (2); although this relationship is still unclear (3, 4). TMD has been characterized by reporting single signs or a combination of signs and symptoms such as orofacial pain, clicking, tenderness, and reduced jaw mobility (5–7). Besides, peripheral factors such as tooth interference in dental occlusion and central or pathophysiological causes involving brain neurotransmitters or basal ganglia, and psychological stress has been implicated in the etiology of bruxism and temporomandibular disorders (8, 9).

COVID-19, commonly known as the novel Coronavirus is believed to have originated from a wet market in Wuhan, China, and has spread all over the world (10). The uncertainty surrounding this pandemic could potentially trigger mental health problems, such as anxiety and depression, in certain subsets of the population (11).

Due to the correlation between stress and bruxism and TMD, so far studies have investigated the prevalence of these disorders in the COVID-19 pandemic. These mostly questionnaire surveys in various populations have shown that social isolation and stressful situations due to the COVID-19 pandemic can increase the number of people with symptoms of TMD, anxiety, and depression.

It could be expected that psychological factors associated to pandemic may lead to a greater risk of developing, worsening and perpetuating bruxism (mainly awake bruxism) and TMD (12, 13). However, Brondani et al. showed that before and after the COVID-19 pandemic, there was no significant change in the prevalence of bruxism (14). Thus, it is unclear that whether or not a relationship between oral habits and psychosocial impacts of COVID-19 pandemic in adults and adolescents do exist.

The present study systematically reviewed the relationship between oral habits (bruxism-TMD) and COVID-19 pandemic in adults and adolescents based on the current literature.

## Materials and Methods

### Study Design

PRISMA guideline served as the framework for this systematic review (15). The PICO question for the study was “Can Coronavirus Disease-19 Lead to oral habits in adults and adolescents?,” detailed as follows: Population: adults and adolescents; Intervention: psychosocial impacts of COVID-19 pandemic; Comparison: N/A; and Outcome: bruxism, TMD.

### Eligibility Criteria

**-Inclusion and Exclusion criteria**

The following papers were included in the study:

- papers assessing the relationship between oral habits and COVID-19 pandemic by questionnaires/interviews
- papers assessing the relationship between TMD symptoms and COVID-19 pandemic by questionnaires/interviews
- papers assessing the relationship between bruxism and COVID-19 pandemic by questionnaires/interviews
- In cases of duplicate studies (i.e., studies presenting the same findings and/or conducted on the same populations), only one article was included for further assessment.
- English language
- full-text papers

Interventional and longitudinal studies in this field were scarce due to the short duration of the disease. Thus, we also included cross-sectional studies in the systematic review.

We excluded review articles, editorials, correspondence, case reports and case series.

### Search Strategy and Study Selection

The following keywords were used: “coronavirus, COVID 2019, SARS2, SARS-CoV-2, severe acute respiratory syndrome coronavirus 2, coronavirus infection, COVID-19, 2019 novel coronavirus disease, SARS-CoV-2 infection, COVID-19 virus disease, 2019 novel coronavirus infection, 2019-nCoV infection, coronavirus disease 2019, coronavirus disease-19, 2019-nCoV, SARS-CoV-2, novel cov, sars cov2, oral habits, parafunction, dysfunction, temporomandibular disorders, craniomandibular disorders, orofacial pain, bruxism.”

We searched PubMed, Scopus, Embase, google scholar, and Cochrane databases. Two authors independently performed an initial search and screening for relevant articles through title and abstract. Discrepancies were resolved by discussion and discretion of the third author. After removal of duplicates, the potential full-texts were evaluated by applying inclusion and exclusion criteria.

### Search

Searches were tailored to the specific databases. An example of a search on PubMed and Cochrane was: (((“SARS-CoV-2”) OR (“COVID-19”) OR (“2019-nCoV”) OR (“coronavirus”)) AND ((“temporomandibular disorders”) OR (“craniomandibular disorders”) OR (“orofacial pain”) OR (“bruxism”) OR (“oral habits”) OR (“parafunction”))).

### Selecting the Articles

**Figure 1:**
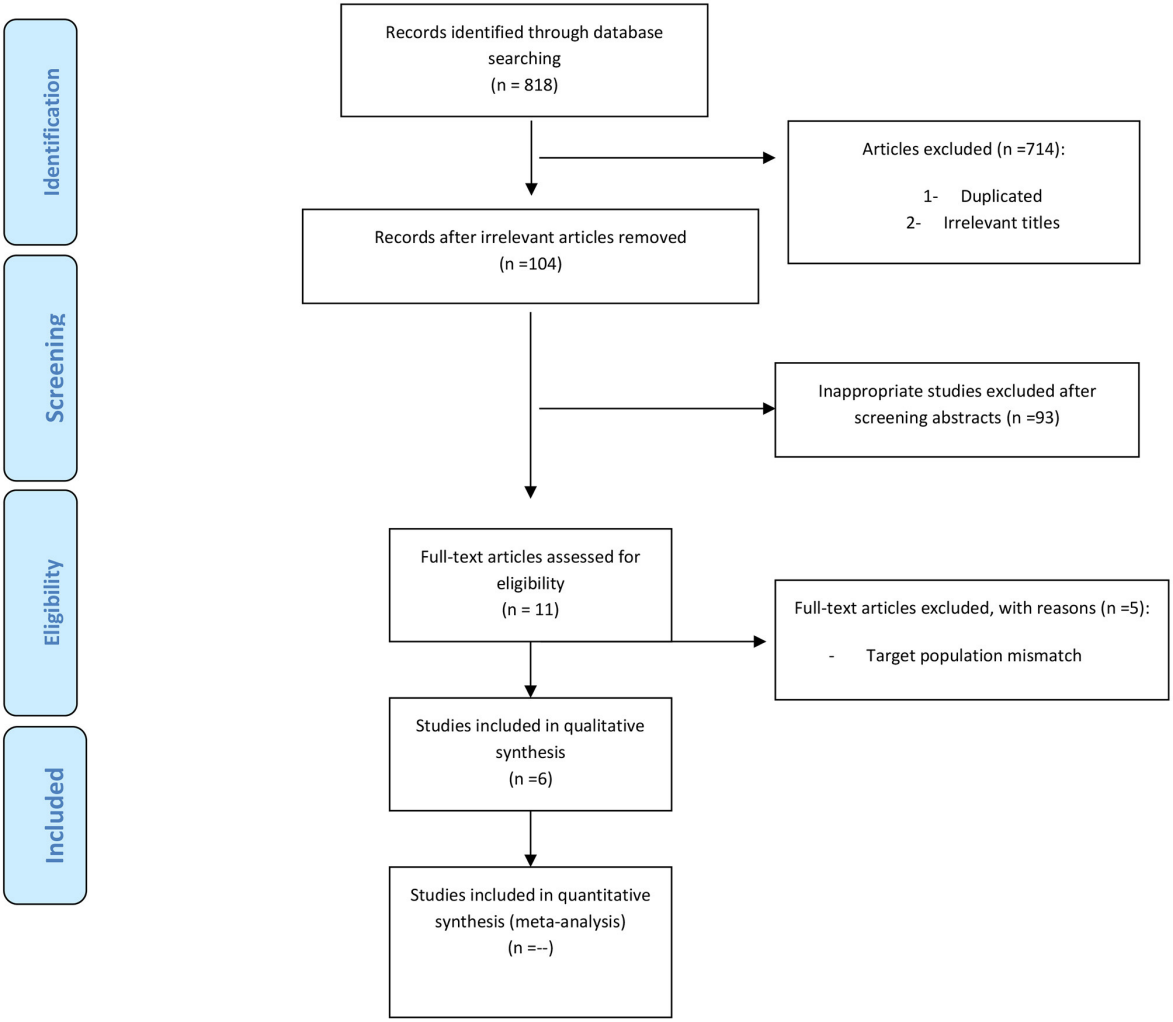
PRISMA flow-chart of selected criteria for the included article reports.

For evaluation of selected articles, titles (independently), abstract and full text of articles was read by two researchers. In case of disagreement between individuals about inclusion or exclusion of article from study, the third researcher read the article. In the initial search 818 articles were obtained; 68 cases were duplicates and excluded. By reviewing the article title, 714 articles were removed because they were not relevant to the topic. Finally, 11 articles were allowed to enter the study. Out of 11 related articles, 5 studies were excluded from the present study due to mismatch of the target population; eventually, 6 full-text articles which met the inclusion criteria were selected for rest of research (Figure 1). In the present study, we ignored other sources of information such as gray literature.

### Extraction and Management of Data

Two independent authors performed data extraction from the studies. We used standardized forms that included author, year, study design, age, gender, evaluation methods and sample size.

The definition of temporomandibular disorder (TMD) used in this review was a variety of conditions affecting the anatomic and functional characteristics of the temporomandibular joint. The definition of bruxism in this review was a disorder characterized by grinding and clenching of the teeth.

### Duplicate Data

The data published several times, was considered a duplicate. To prevent several publications of data, only the original articles were studied. This reduces any overestimation of the effect of the intervention since there are no duplicate data exceptions.

### Investigating the Missing or Defective Data

The strategies for missing/defective data in the present study are as follows:

- Contacting the author if possible.
- Analyzing only the current data (overlooking the missing data).
- Finally, discussing the possible effects of the missing data on the understudy findings in the discussion.

### Risk of Bias

Five of the related studies were excluded from this study due to mismatch of target populations. The study populations in each of these five articles were dental students and patients with temporomandibular joint disorder before the onset of the COVID-19 pandemic. Consequently, six articles were studied and evaluated for bias (Tables 1–3).

**Table 1:** Evaluation of bruxism frequency in studies.

**References**	**Sample size**	**Bruxism**	**Patent with bruxism**	**Evaluation methods**
Brondani et al. (14)	290	Yes	20	Self-report
Carrillo-Diaz et al. (16)	213	Yes	24	Self-report
Wieckiewicz et al. (17)	1,642	Yes	510	OBC (Self- assessment)
Emodi-Perlman et al. (18)	1,792	Yes	772	Self-report
Giacomo et al. (19)	240	Yes	71	Self-report

**Table 2:** Evaluation of TMD (pain) frequency in studies.

**References**	**Sample size**	**TMD (pain)**	**Patent with pain**	**Evaluation methods**
Emodi-Perlman et al. (18)	1,792	Yes	247	3Q.TMD
Saccomanno et al. (20)	182	Yes	75	RDC/TMD^a^
Giacomo et al. (19)	240	Yes	102	RDC/TMD

a *RDC/TMD, Research Diagnostic Criteria for temporomandibular Disorders*.

**Table 3:** Evaluation of TMD (noise) frequency in studies.

**References**	**Sample size**	**TMD (noise)**	**Patent with noise**	**Evaluation methods**
Saccomanno et al. (20)	182	Yes	43	RDC/TMD^\^
Giacomo et al. (19)	240	Yes	100	RDC/TMD

## Results

Heterogeneity in the results of this study was observed due to differences in methodology, particularly in the method of determining outcomes. Meta-analysis was therefore not possible. For this reason, in the discussion section, each study is discussed in terms of inclusion criteria, how outcome variables are measured and the results.

Table 4 summarizes accepted articles.

**Table 4:** Summary of articles.

**References**	**Subject**	**Country**	**Population**	**Age (mean/year)**	**Evaluation methods**		**Outcome**	**Results**
Brondani et al. (14)	Effect of the COVID-19 pandemic on behavioral and psychosocial factors related to oral health in adolescents: a cohort study	Brazil	Brazilian adolescent population	10–12 and 13–15 years	Questionnaire: self-report	—	Bruxism TMD Sleep quality Tooth brushing Sugar consumption Social distance	The COVID-19 pandemic caused significant changes in behavioral and psychosocial factors in adolescents.
Carrillo-Diaz et al. (16)	Lockdown impact on lifestyle and its association with oral parafunctional habits and bruxism in a Spanish adolescent population	Spain	Spanish adolescent population	14 ± 1.9 Years	Questionnaire: Self-report	—-	Bruxism TMD	The adolescent lifestyle has changed during lockdown, with an increase in oral para-functions and bruxism.
Wieckiewicz et al. (17)	Identification of risk groups for mental disorders, headache and oral behaviors in adults during the COVID-19 pandemic	Poland	North American and European population	10–72 years	Questionnaire: HADS(A, D) OBC^a^ MIDAS^b^	- Age - Gender - Race - Relationship status - Education level	Bruxism TMD	During the COVID19 pandemic, both North American and European residents demonstrated an increase in parafunctional oral behaviors.
Emodi-Perlman et al. (18)	Temporomandibular Disorders and Bruxism Outbreak as a Possible Factor of Orofacial Pain Worsening during the COVID-19 Pandemic—Concomitant Research in Two Countries	Israel Poland	Israel and Poland population	Young adults: 18–35 years Adults: 36–56 years	Questionnaire: 3Q.TMD Self-report	- Age - Gender - Race	Bruxism TMD Anxiety	Both Israeli and Polish populations have been affected adversely by the Coronavirus pandemic, resulting in intensification of their bruxism and TMD symptoms.
Saccomanno et al. (20)	Coronavirus Lockdown as a Major Life Stressor: Does It Affect TMD Symptoms?	Italy	Italian population	45 years	Questionnaire: RDC/TMD^c^ PSS^d^	- Age - Gender	TMD Stress Depression	There was a correlation between stress during the pandemic lockdown and the onset of temporomandibular joint disorders and facial pain, although each person's response was different.
Giacomo et al. (19)	Psychological impact of COVID-19 pandemic on TMD subjects	Italy	Italian population	28 years	Questionnaire: RDC/TMD PSS	- Age - Gender	Bruxism TMD Sleep quality Stress	During the pandemic, parafunctions increased.

a *OBC, Oral behavior checklist*.

b *MIDAS, Migraine disability assessment*.

c *RDC/TMD, Research Diagnostic Criteria for temporomandibular Disorders*.

d *PSS, Perceived Stress Scale*.

Brondani et al. evaluated the impact of the COVID-19 pandemic on behavioral and psychosocial aspects related to oral health. Based on this study, behavioral and psychosocial factors changed significantly in adolescents due to the COVID-19 pandemic (14).

Carrillo-Diaz et al. investigate the relationship between a decrease in physical and social activity and an increase in the use of mobile devices, the internet, and social media. There was an increase in anxiety and the appearance of oral para-functions and bruxism for adolescents during COVID-19 pandemic; based on their data, the increase in social network use at night and anxiety levels during lockdown appear to be associated with an increase in self-reported bruxism (16).

Wieckiewicz et al. identify predictors, risk factors, and factors related to mental disorders, headaches, and potentially stress-modulated para-functional oral behaviors related to the COVID-19 pandemic in North America and Europe. The results from this study enabled the definition of the group most likely to experience these indirect health effects of the pandemic. This group included younger females than 28, especially those who were single, less educated, and living in Europe (17).

Emodi-Perlman et al. investigated the impact of the current pandemic on TMD and bruxism prevalence and symptoms among participants from two culturally different countries (Israel and Poland). In both Israeli and Polish populations, the COVID-19 pandemic has resulted in an increase in bruxism and TMD symptoms resulted from the psychoemotional distress caused by the pandemic (18).

Saccomanno et al. examined the presence of TMDs, the sudden onset of these symptoms, and the manner in which these symptoms worsened in light of social changes that resulted from the recent pandemic induced by a coronavirus. According to the findings of this study, stress during the pandemic lockdown influenced the onset of TMDs and facial pain (20).

Di Giacomo et al. assessed the psychological impact of the COVID-19 pandemic on those with temporomandibular disorders (TMD) as well as for symptomatology and parafunctionality; they found that awake and sleep bruxism, dental grinding, and sleep disruption, as well as fatigue, increased during the pandemic (19).

## Discussion

The present study reviewed and evaluated the relationship between oral habits (bruxism- TMD) and COVID-19 pandemic in adults and adolescents. Studies have shown that stress caused by the COVID-19 pandemic increases detrimental oral habits such as bruxism as well as temporomandibular disorders in adults and adolescents; In general, young single women are at high risk and more exposed to these harmful oral habits. Bruxism is a destructive oral habit that is defined as the non-productive diurnal or nocturnal clenching or grinding of the teeth which can also lead to TMD (21). There are many scientific reports about the coexistence of bruxism, stress, and psychoemotional disorders (22, 23). In the last 2 years, we have faced a COVID-19 pandemic that has brought a lot of psychological stress to human beings, and in addition to the physical consequences, we must pay attention to the psychosocial impacts of COVID-19 pandemic.

In a study, Saccomanno et al. assessed the incidence of bruxism and TMD in various age ranges during the COVID-19 pandemic, reporting an increase in the incidence of the mentioned conditions. In addition, two age ranges of 30–40 and 50–60 years were identified as more vulnerable age groups regarding the incidence of bruxism and TMD. They evaluated the prevalence of stress and depression, declaring that the two parameters had a higher increase in the aforementioned age groups during the COVID-19 pandemic (20). According to Emodi-Perlman et al. while bruxism and anxiety increased in the COVID-19 pandemic, their incidence rates were higher in the age group of 36–55 years (18). Wieckiewicz et al. reported a high TMD incidence rate in females younger than 28.5 years and males in the age range of 30–34 years during the COVID-19 pandemic. These two age groups are more present in the community and interact with different people, which has been associated with risks such as person-to-person transmission during COVID-19. Meanwhile, the psychological effects of this issue cannot be overlooked (17). Some studies have assessed the relationship between increased oral health habits (e.g., bruxism and TMD) and gender, race and marital status of individuals in addition to approving the increased prevalence of these issues in age groups of adolescents and adults.

Brondani et al. evaluated behavioral and psychosocial factors related to oral health in adolescents in two periods before and after COVID-19 pandemic. According to their results, behavioral and psychosocial factors significantly changed in adolescents due to the pandemic. In addition, they detected a significant decrease in the frequency of brushing and visit to dental clinic (14). A decrease was also observed in the frequency of sugar consumption, the prevalence of bruxism and sleep quality after COVID-19 pandemic, compared to before COVID-19 pandemic, which was not significant. While stress is expected to increase bruxism, it actually decreased bruxism in the foregoing research. To explain this reduction, they mentioned two reasons: first, age group; their subjects were adolescents, who had less symptoms of depression and sleep disorders, compared to adults, and were less concerned with the prevailing situation in the society; and second, self-reported nature of the study, which may lead to underestimation of bruxism prevalence (14).

In contrast, Carrillo-Diaz et al. in Spain studied the prevalence of para-functional habits and bruxism in teenagers in two time periods before the COVID-19 pandemic and after the COVID-19 pandemic. They also indicated an increase in the outbreak of bruxism after COVID-19 pandemic compared to that before COVID-19 pandemic due to stress originating from the Corona pandemic. Also, they implemented self-reported method to assess bruxism (16). The controversy between the results of these two studies might be related to race. The level of stress and the outbreak of related symptoms can differ from race to race. According to Wieckiewicz et al. the outbreak of bruxism and temporomandibular disorders (TMD) during the Corona pandemic in Americans was significantly higher than in Europeans (17), and in the Emodi-Perlman study in Polish people was noticeably higher than in Israelis. Studies have indicated a higher outbreak of TMD and bruxism in women (18). According to the studies, the female gender is a significant factor in TMD and bruxism's prevalence (24, 25). According to recent studies, gender affects the anxiety level and women encounter mood disorders almost twice as much as men. Also, studies indicated that early-onset generalized anxiety disorder (GAD) is related to the female gender, and the level of stress and anxiety is higher in women (26, 27). Recent studies have also indicated that the outbreak of stress, depression, and anxiety during the Corona pandemic is higher in women than men (28). Therefore, we can assume that women are more prone (susceptible) to damaging oral habits, such as bruxism, followed by TMD symptoms, regarding the stress experienced during the period of the Corona pandemic. Wieckiewicz et al. showed that the Hospital Anxiety and Depression Scale (HADS-D) in the unmarried group was higher than in the married group. Other factors such as oral habits (TMD and bruxism) were higher in the married group (10.28 ± 23.41) than the unmarried group (22.77 ± 10.54). The reason for the increase in para-functional habits in alone and unmarried people is that they feel more depression in their life. But in married life, there is more anxiety due to living with more people, with different occupations and different systemic health, which can increase the risk of bruxism and TMD symptoms (17).

## Conclusion

Studies have shown that stress caused by the COVID-19 pandemic increases oral habits such as bruxism as well as temporomandibular disorders in adults and adolescents. This review study may help identify sensitive and high-risk groups by evaluating various studies. In general, young single women are at a higher risk and exposed to these harmful oral habits.

## Data Availability Statement

The original contributions presented in the study are included in the article/supplementary material, further inquiries can be directed to the corresponding authors.

## Author Contributions

AM: contributed to the study design, guidance throughout the study, and manuscript editing. MKham: contributed to performing the study and revising the article. MKhar: contributed to performing the study. RB: contributed to performing the study, study design, data analysis, and writing the manuscript. All authors contributed to the article and approved the submitted version.

## Conflict of Interest

The authors declare that the research was conducted in the absence of any commercial or financial relationships that could be construed as a potential conflict of interest.

## Publisher's Note

All claims expressed in this article are solely those of the authors and do not necessarily represent those of their affiliated organizations, or those of the publisher, the editors and the reviewers. Any product that may be evaluated in this article, or claim that may be made by its manufacturer, is not guaranteed or endorsed by the publisher.
